# Force Sensitivity in *Saccharomyces cerevisiae* Flocculins

**DOI:** 10.1128/mSphere.00128-16

**Published:** 2016-08-17

**Authors:** Cho X. J. Chan, Sofiane El-Kirat-Chatel, Ivor G. Joseph, Desmond N. Jackson, Caleen B. Ramsook, Yves F. Dufrêne, Peter N. Lipke

**Affiliations:** a Haskins Laboratories and Department of Chemistry and Physical Sciences, Pace University, New York, New York, USA; b Université Catholique de Louvain, Institute of Life Sciences, Louvain-la-Neuve, Belgium; c Biology Department, Brooklyn College, City University of New York, New York, New York, USA; University of Texas Health Science Center

**Keywords:** biofilms, functional amyloid, fungal adhesins, glycoproteins

## Abstract

Many fungal adhesins have short, β-aggregation-prone sequences that play important functional roles, and in the *Candida albicans* adhesin Als5p, these sequences cluster the adhesins after exposure to shear force. Here, we report that *Saccharomyces cerevisiae* flocculins Flo11p and Flo1p have similar β-aggregation-prone sequences and are similarly stimulated by shear force, despite being nonhomologous. Shear from vortex mixing induced the formation of small flocs in cells expressing either adhesin. After the addition of Ca^2+^, yeast cells from vortex-sheared populations showed greatly enhanced flocculation and displayed more pronounced thioflavin-bright surface nanodomains. At high concentrations, amyloidophilic dyes inhibited Flo1p- and Flo11p-mediated agar invasion and the shear-induced increase in flocculation. Consistent with these results, atomic force microscopy of Flo11p showed successive force-distance peaks characteristic of sequentially unfolding tandem repeat domains, like Flo1p and Als5p. Flo11p-expressing cells bound together through homophilic interactions with adhesion forces of up to 700 pN and rupture lengths of up to 600 nm. These results are consistent with the potentiation of yeast flocculation by shear-induced formation of high-avidity domains of clustered adhesins at the cell surface, similar to the activation of *Candida albicans* adhesin Als5p. Thus, yeast adhesins from three independent gene families use similar force-dependent interactions to drive cell adhesion.

**IMPORTANCE** The *Saccharomyces cerevisiae* flocculins mediate the formation of cellular aggregates and biofilm-like mats, useful in clearing yeast from fermentations. An important property of fungal adhesion proteins, including flocculins, is the ability to form catch bonds, i.e., bonds that strengthen under tension. This strengthening is based, at least in part, on increased avidity of binding due to clustering of adhesins in cell surface nanodomains. This clustering depends on amyloid-like β-aggregation of short amino acid sequences in the adhesins. In *Candida albicans* adhesin Als5, shear stress from vortex mixing can unfold part of the protein to expose aggregation-prone sequences, and then adhesins aggregate into nanodomains. We therefore tested whether shear stress from mixing can increase flocculation activity by potentiating similar protein remodeling and aggregation in the flocculins. The results demonstrate the applicability of the Als adhesin model and provide a rational framework for the enhancement or inhibition of flocculation in industrial applications.

## Abstract (precis)

The *Saccharomyces cerevisiae* flocculins mediate the formation of cellular aggregates and biofilm-like mats, useful in clearing yeast from fermentations. An important property of fungal adhesion proteins, including flocculins, is the ability to form catch bonds, i.e., bonds that strengthen under tension. This strengthening is based, at least in part, on increased avidity of binding due to clustering of adhesins in cell surface nanodomains. This clustering depends on amyloid-like β-aggregation of short amino acid sequences in the adhesins. In *Candida albicans* adhesin Als5, shear stress from vortex mixing can unfold part of the protein to expose aggregation-prone sequences, and then adhesins aggregate into nanodomains. We therefore tested whether shear stress from mixing can increase flocculation activity by potentiating similar protein remodeling and aggregation in the flocculins. The results demonstrate the applicability of the Als adhesin model and provide a rational framework for the enhancement or inhibition of flocculation in industrial applications.

## INTRODUCTION

Yeast cell surface adhesins mediate cell-to-cell aggregation and cell-to-surface adhesion. Among the adhesins, the *Saccharomyces cerevisiae* flocculins encompass several members of the Flo1 family, i.e., Flo1p, Flo5p, Flo9p, and Flo10p, as well as an unrelated flocculin, Flo11p (previously named Muc1p). These proteins mediate Ca^2+^-dependent aggregation of cells (1, 2). In fermentations, expression of Flo1p or its paralogs late in the process can aggregate the yeast and help clarify the product (3, 4).

Flocculins also confer the ability to bind to and invade agar (2). In the wild, flocculins mediate the formation of biofilm-like mats. Flo1p acts as a “greenbeard gene” that allows yeast cells to recognize and coaggregate with other Flo1p-expressing cells to form toxin-resistant mats (5). Flo1p is also shed from cells to form a matrix in these mats (6). Flo11p is present in many wild strains: some alleles mediate flocculation, and some mediate the formation of “flor,” a liquid-aerial surface biofilm important in the fermentation of sherry (7). Because Flo11p also mediates the formation of cell aggregates and mats and its expression increases under stress, it may be a primary means of adaptation to stress (8).

**FIG 1:**
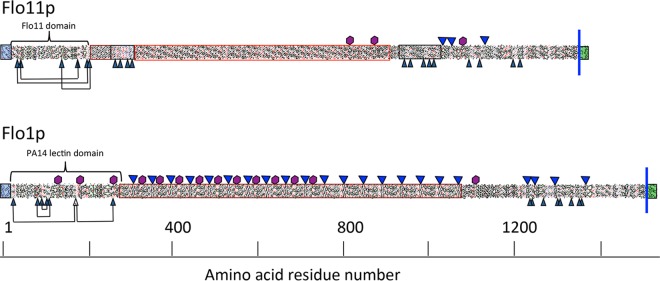
Primary-structure analyses of Flo1p and Flo11p. Hydrophobic-cluster analyses highlight domain structure and patterns of repeats (15). Secretion signal sequences are boxed in blue, C-terminal GPI addition signals are boxed in green, and the blue line denotes the position of GPI signal cleavage and anchorage to cell wall glucan (13). Central repeat regions are in unshaded boxes. Cys residues are black arrowheads, with disulfides marked where they have been mapped in the N-terminal domains (12). Potential N-glycosylation sites are marked with purple hexagons, and sequences with a β-aggregation potential of >30% in TANGO are marked with blue triangles (21).

Although many yeast adhesins are nonhomologous, they share commonalities in overall structure and in the presence of potential β-aggregation-prone sequences. They are similar in architecture, with an N-terminal secretion signal sequence, a β-sheet-rich globular ligand-binding region (9–12), a midregion containing serine/threonine-rich tandem repeats (TRs), a long Ser/Thr-rich glycosylated stalk, and a C-terminal glycosylphosphatidylinositol (GPI) anchor (13). During cell wall biogenesis, the GPI anchor is cleaved in the glycan and the remnant is covalently attached to cell wall polysaccharide (14). These features of Flo1p and Flo11p are illustrated in Fig. 1 as HCA drawings that emphasize repeat patterns, hydrophobic regions (green), Cys residues, and N-glycosylation sites (15).

These similarities lead to similar responses to extension force in atomic force microscopy (AFM). *Candida albicans* Als proteins and *S. cerevisiae* Flo1p show force-distance curves characteristic of successive unfolding of the TRs and other domains, with total extension lengths of up to 300 nm. Cell-cell adhesion in AFM thus shows similar patterns, with longer extension lengths due to stretching of pairs of adhesins extending for each cell surface (16–18). Force response of Flo11p has not been previously reported.

Within these adhesins are five- to seven-amino-acid sequences predicted by TANGO (http://tango.crg.es/) to form β-aggregate structures (19–21). These sequences are especially rich in the β-branched aliphatic amino acids Ile, Val, and Thr. In Flo1p, each of 18 TRs contains a predicted β-aggregating sequence, and there are four more TANGO-positive sequences in the C-terminal region. A peptide containing a Flo1p TANGO-positive sequence forms amyloid fibers: TDETVIVIRTP (β-aggregation-positive residues are underlined) (20). There are three TANGO-positive sequences in Flo11p, and a 1,331-residue soluble version of the protein assembles into amyloid fibers. Anti-amyloid compounds inhibit flocculation due to Flo1p or Flo11p, and the surfaces of cells expressing these flocculins are birefringent and bind the amyloid-sensitive dye thioflavin T (ThT) (20). These results are consistent with the hypothesis that β-aggregated flocculins participate in cell-cell interactions (22).

We have recently proposed that extension forces expose β-aggregation-prone sequences in the adhesin Als5p from *C. albicans*, and this exposure is followed by aggregation into high-avidity adhesion patches on the cell surface. Consequently, clustering activates Als protein-mediated adhesion (23, 24). The activation is triggered by extension forces including single-molecule stretching in AFM, laminar flow, or vortex mixing of the cells (24–26). The resulting surface nanodomains are 100 to 500 nm in diameter and ThT bright (22, 24). In cells expressing Als5p, extension force leads to nanodomain formation within 5 to 15 min and the nanodomains propagate slowly around the cell surface at a rate of ~20 nm/min (23).

These observations lead to the question of whether force-dependent activation of nanodomain formation is general or specific to Als5p. Specifically, the *S. cerevisiae* flocculins have TANGO-positive sequences, and anti-amyloid compounds inhibit flocculation (20). Therefore, we tested whether hydrodynamic shear also leads to the formation of surface nanodomains and potentiation of cell-cell aggregation.

## RESULTS

### β-Aggregation-positive sequences in flocculins.

The protein sequences of Flo1p and Flo11p were analyzed with Draw HCA and the β-aggregation predictor TANGO (Fig. 1; see Table S1 in the supplemental material) (21, 27, 28). Most of the primary structures consist of TRs, as illustrated by the repeating patterns in the HCA profiles (Fig. 1; outlined with uncolored boxes). Of the 22 sequences in Flo1p with β-aggregation potential values of >30%, 18 conform to the consensus T(V/I)IVI; each of these is in a 45-residue repeat. These predicted β-aggregation sequences are rich in β-branched aliphatic amino acids Ile, Thr, and Val, similar to the β-aggregation sequence of Als5p. These sequences are also positive in Aggrescan and ZipperDB, but only one (position 1299 in Flo1, TVVTI) is also positive in WALTZ (29). In contrast, the three TANGO-positive sequences of Flo11p are found after residue 1000 only. Aggrescan analysis showed 18 aggregation-prone sequences in this C-terminal region, including the three β-aggregation sequences (30).

10.1128/mSphere.00128-16.3 Table S1  β-Aggregation-positive sequences of *S. cerevisiae* adhesins. TANGO was used to predict the potential β-aggregate-forming sequences in Flo1p and Flo11p. The settings were a pH of 5.5, a temperature of 298.15 K, an ionic strength of 0.02, and no N- or C-terminal protection. Five-hundred-residue segments were scanned through TANGO with 100-residue overlaps. The Flo1p NCBI accession number is NP_009424. The Flo11p GenBank accession no. is ABS87372.1. Sequences predicted by TANGO to have β-aggregation potential values of >30% are included. Download Table S1, PDF file, 0.1 MB. Copyright © 2016 Chan et al. 2016 Chan et al. This content is distributed under the terms of the Creative Commons Attribution 4.0 International license.

### Effects of hydrodynamic shear on flocculation of Flo1p- and Flo11p-expressing *S. cerevisiae* cells.

**FIG 2:**
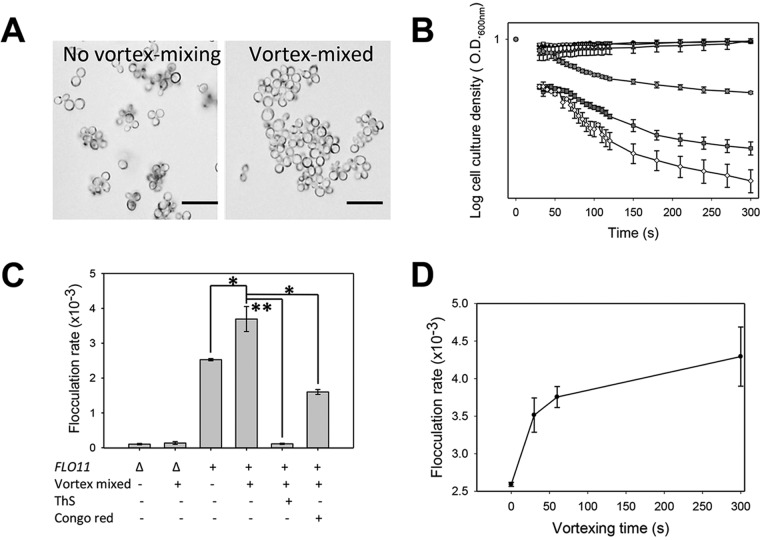
Effects of vortex mixing on the extent and rate of flocculation of *S. cerevisiae* var. *diastaticus* cells expressing Flo11p. (A) Cells were left untouched or vortex mixed and then visualized in the absence of Ca^2+^. Scale bars = 20 µm. (B) Flocculation assays after the addition of 330 µM Ca^2+^. Cells assayed: Δ*flo11* mutant not mixed (black circles, top) or vortex mixed for 5 min (inverted triangles, top); *FLO11* cells not mixed (dark gray boxes) or vortex mixed in the absence of inhibitors (light-shaded diamonds) or in the presence of 200 µM ThS (dark triangles, top) or 500 µM CR (gray circles). Each error bar shows the standard error of the mean of three or more values. (C) Inhibition by ThS (200 µM) and CR (500 µM) (*n* = 3). Flocculation rates were calculated between 40 and 80 s. *, *P* < 0.05; **, *P* < 0.001 (Student’s *t* test). (D) Effect of the duration of vortex mixing on the flocculation rate. Each error bar shows the standard error of the mean of three values.

**FIG 3:**
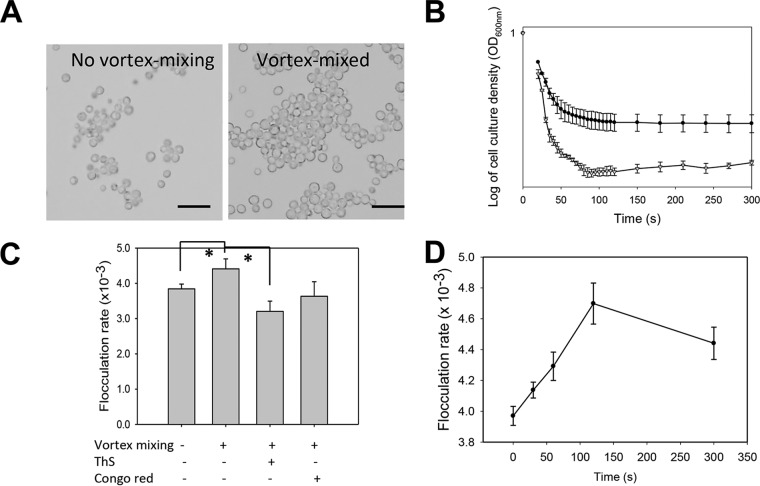
Effects of vortex mixing on the flocculation of *S. cerevisiae* cells expressing Flo1p. (A) Cells were left untouched or vortex mixed and then visualized in the absence of Ca^2+^. Scale bars = 20 µm. (B) Flocculation assays after the addition of 1.0 mM Ca^2+^. Cells were not mixed (●) or vortex mixed for 5 min (▽). (C) Flocculation rates in the absence or presence of the inhibitors ThS (200 µM) and CR (0.5 mM) (*n* = 3). *, *P* < 0.01 (Student’s *t* test). (D) Effect of duration of vortex mixing on activation of flocculation.

We tested whether the flocculins were force activated in a manner similar to that of Als5p (31). *S. cerevisiae* var. *diastaticus* cells express Flo11p naturally. Cell suspensions in buffer with EDTA were left untouched or sheared by vortex mixing for 5 min and then inspected for the presence of flocs. The cells that were not vortex mixed had mean floc sizes of five to seven cells, and vortex mixing increased the mean number of cells per floc 9-fold, as determined by image analysis of three microscopic fields (Fig. 2A). Ca^2+^ was added to initiate flocculation, and the reaction was monitored by optical densitometry (Fig. 2B) (2, 32). Both the rate of flocculation and the number of flocculating cells were greater in populations that had been vortex mixed (Fig. 2B and C). Specifically, Flo11p-expressing cells had flocculation rates 1.5-fold higher than those of cells that had not been vortex mixed. Thirty seconds of mixing increased the flocculation rate 1.3-fold, from (2.6 ± 0.04) × 10^−3^ to (3.5 ± 0.4) × 10^−3^. The rate increased to 4.2 × 10^−3^ with 5 min of mixing (Fig. 2D). This timing was similar to that for the adhesin Als5p from *C. albicans* (31). An *S. cerevisiae* var. *diastaticus* mutant strain with *flo11* deleted did not flocculate (2) and had no increase in flocculation when vortex mixed (Fig. 2B and C), so there was apparently little expression of compensatory adhesins such as Flo1p (Fig. 2B and C). We also tested *S. cerevisiae* expressing Flo1p. Vortex-induced shearing in EDTA-containing buffer increased the size of initial flocs 80-fold over the mean of three or four cells per floc in quiescent cell suspensions (Fig. 3A). When Ca^2+^ was added, the rate and extent of flocculation both increased greatly (Fig. 3B and C). There was significant activation of Flo1p-expressing cells after 30 s of vortex mixing and maximum activation at 120 s (Fig. 3D).

### Effects of amyloid-binding dyes on flocculation and agar invasion.

Thioflavin S (ThS; 200 µM) and Congo red (CR; 500 µM) inhibit the flocculation of Flo11p- and Flo1p-expressing cells (20). We therefore determined whether these dyes would inhibit the increase in the rate and extent of flocculation of Flo11p-expressing cells that were vortex mixed. Flo11p-expressing cells treated with 500 µM CR had a 2.3-fold decrease in the flocculation rate, and cells treated with 200 µM ThS had a 33-fold decrease in the flocculation rate (Fig. 2B and C). Similarly, CR and ThS also inhibited force-activated flocculation in Flo1p-expressing cells (Fig. 3C).

**FIG 4:**
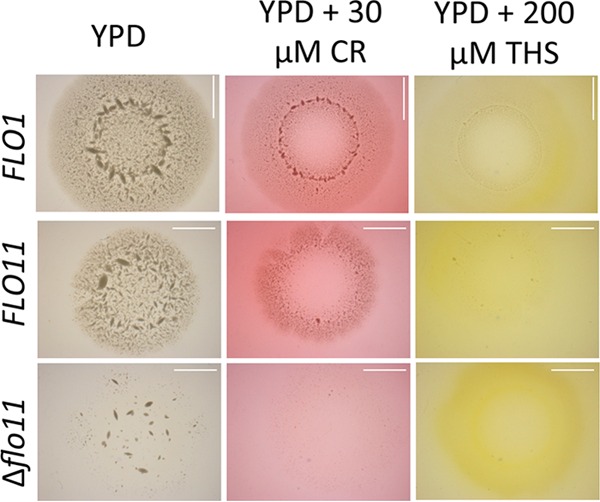
Agar invasion assays. Cells were grown on YPD plates for 2 weeks with or without amyloidophilic dyes and then washed and imaged from the bottom of the petri plate. Concentrations: CR, 30 µM; ThS, 200 µM. Scale bars represent 5 mm.

Expression of flocculins also confers on yeast the ability to invade agar (2), although Flo11p from *S. cerevisiae* var. *diastaticus* is not as active as other Flo11p isoforms (1, 13, 34). We therefore tested whether this activity is also inhibitable by amyloid-binding dyes. Agar plates were prepared with or without 30 µM CR and/or 200 µM ThS. Colonies were grown for 2 weeks and then washed off under a stream of water. The microcolonies of cells that had invaded the agar were imaged from the bottom of the plate (1, 35). Flo1p or Flo11p mediated invasive inclusions in the agar, with small dense colonies and a diffuse halo (Fig. 4, left column). A Δ*flo11* mutant formed a few small inclusions. In the presence of CR, the size and number of invasion inclusions decreased and the included colonies were smaller. ThS was even more potent in preventing invasion; no strains showed any inclusions. Thus, CR and ThS inhibited the inclusions of yeast cells in the agar.

### Effect of vortex mixing on cell surface ThT fluorescence.

**FIG 5:**
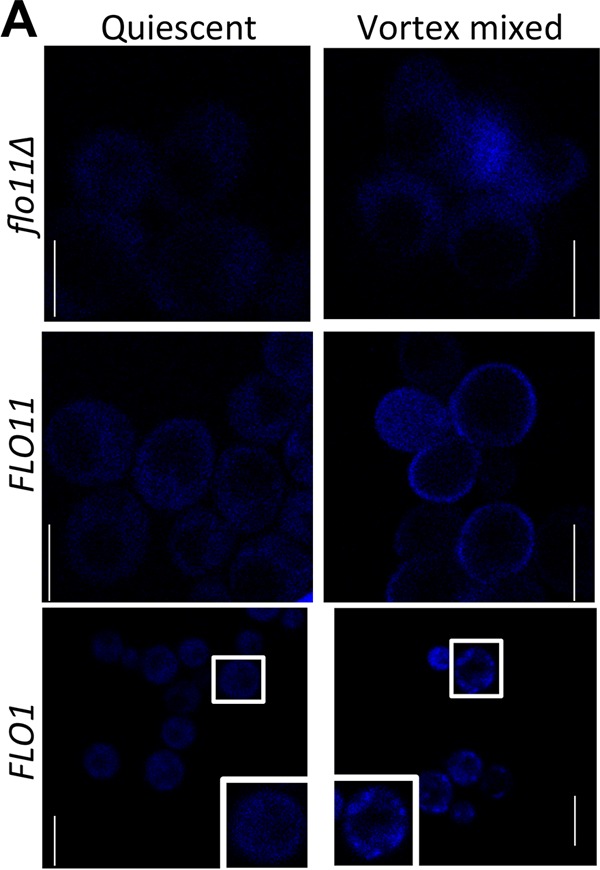
ThT fluorescence on the surface of Flo11p- and Flo1p-expressing *S. cerevisiae*. Shown are fluorescence confocal micrographs of cells under quiescent and vortex-mixed conditions taken right after vortex mixing as described in Materials and Methods and then stained with 500 nM ThT. Scale bars represent 5 µm. The boxed cells are further enlarged in the insets.

Because extension of individual Als5p adhesin molecules leads to the formation of thioflavin-bright surface nanodomains, we tested whether there were similar responses in the flocculins (20, 23, 24, 36, 37). Flo1p- or Flo11p-expressing cells were vortex mixed for 5 min and then stained with ThT (500 nM), which has less inhibitory activity than ThS. The results showed increased density and fluorescence of ThT-stained nanodomains on cells expressing Flo1p or Flo11p (Fig. 5). This increase was not seen with the *S. cerevisiae* var. *diastaticus* strain with a deletion in *flo11*. In Flo11p- and Flo1p-expressing cells, vortex mixing also increased the mean ThT fluorescence (Table 1; see Fig. S1 in the supplemental material). For Flo11p-expressing cells, the mean fluorescence intensity of cells increased 31% after vortex mixing. Flo1p-expressing cells showed a 55% increase in mean fluorescence. Therefore, vortex mixing of cells expressing any of the three adhesins led to significant increases in surface fluorescence with ThT, and these increases accompanied increased flocculation activity.

10.1128/mSphere.00128-16.1 Figure S1  Overlays of ThS fluorescence of quiescent cell populations (black) or cells vortex mixed for 5 min (gray). (A) Flo1-expressing cells. (B) Flo11-expressing cells. Download Figure S1, PDF file, 0.04 MB. Copyright © 2016 Chan et al. 2016 Chan et al. This content is distributed under the terms of the Creative Commons Attribution 4.0 International license.

**Table 1:** Effect of vortex mixing on the mean ThT fluorescence of cells expressing adhesins^a^

Expressed adhesin	Cell fluorescence		Ratio
	Quiescent	Vortex mixed	
None (EV)	142	161	1.13
ΔFLO11	987	994	1.01
FLO11	864	1,128	1.31
FLO1	889	1,367	1.55

a Cells of each type were vortex mixed for 5 min at 2,500 rpm or left quiescent for 5 min. The cells were then stained with 500 nM ThT and assayed by flow cytometry.

Activation of Als5p nanodomains is cell autonomous in the sense that dead cells also exhibit the phenomenon (31). Similarly, vortex mixing activated flocculation in heat-killed cells expressing either Flo1p or Flo11p in the presence of Ca^2+^ (see Fig. S2 in the supplemental material).

10.1128/mSphere.00128-16.2 Figure S2  Effects of vortex mixing on heat-killed flocculin-expressing cells. (A) Effects of vortex mixing on the flocculation rate of Flo11p-expressing cells after heat killing. (B) Effects of vortex mixing on the flocculation rate of Flo1p-expressing cells after heat killing. Error bars represent the standard deviation. Download Figure S2, PDF file, 0.1 MB. Copyright © 2016 Chan et al. 2016 Chan et al. This content is distributed under the terms of the Creative Commons Attribution 4.0 International license.

### Single-molecule analysis demonstrates the localization and mechanics of single Flo11p.

**FIG 6:**
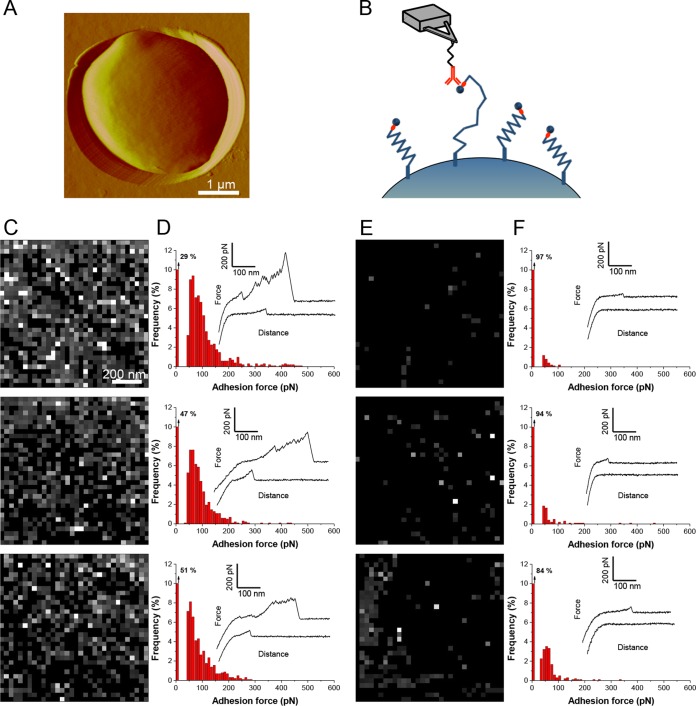
Single-molecule imaging of V5-Flo11p proteins on yeast cells. (A) AFM deflection image recorded in buffer, showing an *S. cerevisiae* cell expressing V5-tagged Flo11p proteins trapped in a porous membrane for AFM analysis. (B) Single-molecule detection of V5-tagged Flo11p proteins with anti-V5 antibody-coated AFM tips. (C to F) Adhesion force maps (1 by 1 µm) (C, E) and adhesion force histograms (*n* = 1,024 curves) (D, F), with representative force curves, obtained in buffer between anti-V5 tips and three different yeast cells expressing V5-tagged Flo11p (C, D) or no flocculin (E, F) (different cultures and different tips). Pixels are shaded according to the rupture force necessary to disrupt binding, with no binding depicted as black and strong interactions shown as white.

We used AFM (38, 39) to further investigate the role of Flo11p in yeast flocculation. First, single Flo11p proteins were mapped and functionally analyzed on live cells by single-molecule force spectroscopy (Fig. 6). For specific detection, a V5 epitope tag was inserted at the N-terminal end of the proteins and the AFM tips were functionalized with anti-V5 antibodies (Fig. 6) (23). The host strain, W303-1B, does not express other flocculins, in part because of a partial deletion in *FLO8*, an activator of transcription for *FLO1* (13). Yeast cells expressing V5-tagged proteins were immobilized in porous polymer membranes (Fig. 6A). Figure 6C shows force maps and the adhesion force histogram with representative force curves recorded between the antibody tips and the surfaces of three different yeast cells expressing V5-Flo11p. A large proportion (49 to 71%) of the force curves showed adhesion signatures reflecting the detection of single Flo11p proteins. There were some dark areas in Fig. 6C and in comparable maps for Flo1p (17), results that suggest a nonrandom cell surface distribution. The high frequency of V5-Flo11p detection corresponded to a minimum protein surface density of ~500 to 730 protein molecules/µm^2^ (assuming only a single molecule per pixel), thus showing that the adhesin was massively exposed compared to Als5p (23).

The force curves were of two different shapes, i.e., low adhesion force curves (Fig. 6C, lower curves) with single small adhesion forces and high adhesion force curves (Fig. 6C, upper curves) with sawtooth patterns with multiple large-force peaks, characteristic of individual domains unfolding sequentially (17, 26). Previous data obtained for Als adhesins revealed that the low-force peaks correspond to the weak molecular recognition of specific V5 epitopes in the tagged adhesin (23), while the high-force peaks represent strong multipoint attachment of the adhesin to the antibody mounted on the tip, enabling the sequential unfolding of the TR domains upon pulling of the tip (17, 23). These signatures were essentially missing from yeast cells lacking Flo11p (empty vector [EV], Fig. 6E and F), leading us to believe that they are specific to Flo11p. Given the structural similarities between the Flo and Als proteins, it is very likely that they show similar biophysical properties when being stretched.

### Single-cell analysis demonstrates that Flo11p mediates cell-cell adhesion via homophilic interactions.

**FIG 7:**
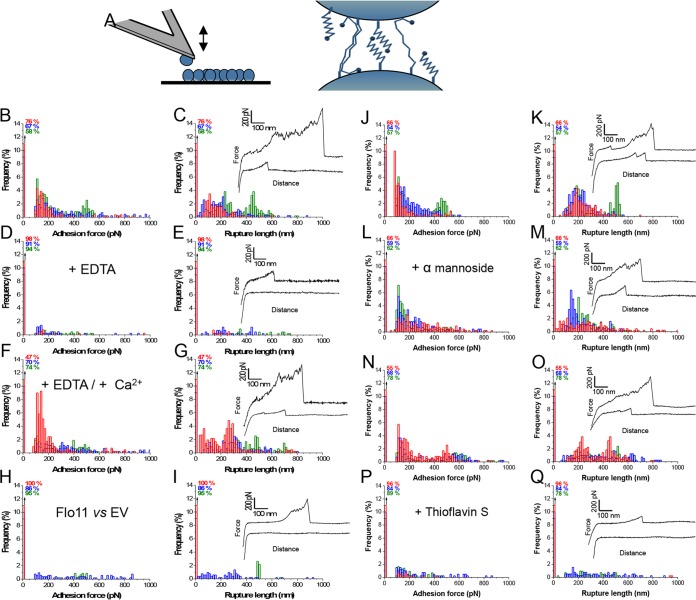
Forces in Flo11p-mediated cell-cell adhesion. (A) Principle of single-cell force spectroscopy. (Left) Single yeast cells were attached to a polydopamine-coated tipless cantilever, and curves of force between cell probes and yeast aggregates were acquired. (Right) Cartoon of homotypic interactions of the flocculins. Shown are adhesion force histograms (B, D, F, H, J, L, N, P) and rupture length histograms (C, E, G, I, K, M, O, Q) with representative force curves obtained by recording multiple force-distance curves for Flo11p-expressing cells in acetate buffer containing 200 µM CaCl_2_ (B, C) or after the addition of 10 mM EDTA (D, E) and further addition of 400 µM CaCl_2_ (F, G). (H, I) Force data obtained with Flo11p-expressing cells and yeast cells expressing no flocculin (EV). (L, M) Effects of methyl alpha-d-mannopyranoside at 40 mg/ml added to the buffer in the experiments shown in panels J and K. For those shown in panels P and Q, 200 µM ThS was added to the cells shown in panels N and O. Red, blue, and green represent results from three different cell pairs from independent cultures (*n* = >400 curves for each cell). All curves were obtained at 20°C by a constant approach at a retraction speed of 1 µm ⋅ s^−1^ with a 1-s contact time.

Next, we measured the forces engaged in Flo11-mediated cell-cell adhesion. To this end, force-distance curves were recorded between cell probes and small cell aggregates adhering to solid substrates by means of single-cell force spectroscopy (Fig. 7A). Figure 7B and C show the adhesion force histograms and rupture length histograms, together with representative force curves, recorded in three different pairs of Flo11p cells. Many curves (24 to 42%) showed adhesion peaks with a 100- to 700-pN magnitude and a 50- to 600-nm rupture length (*n* = >1,200 curves from three independent cell pairs). As with single-Flo11p experiments, force profiles showed either single adhesion peaks with low force or multiple large adhesion peaks attributed to the force-induced unfolding of the TR domains of Flo11p. Figure 7D to G show that cell-cell adhesion forces were abolished in the presence of EDTA and restored upon the further injection of Ca^2+^, implying that Flo11p interactions are calcium dependent, like Flo1p (17). However, unlike with Flo1p cells, addition of free methyl alpha-d-mannopyranoside had no substantial effect on Flo11p interaction forces, so the binding did not involve a mannose-dependent lectin-like mechanism (Fig. 7J to M). Our finding that the forces between Flo11p-expressing cells and cells without Flo11p were very weak implies that the bonds were homophilic (Fig. 7H and I). Lastly, adhesion forces were abolished by the addition of ThS (Fig. 7N to Q). Accordingly, our single-cell and single-molecule analyses show that Flo11p adhesion involves homophilic interactions between flocculins localized on neighboring cells.

## DISCUSSION

Our results show that unrelated fungal adhesins can have similar responses to shear force, consistent with similarities in their potential β-aggregation sequences. Flo1p and Flo11p of *S. cerevisiae* are like the *C. albicans* adhesin Als5p in that they are clustered on the cell surface in arrays and cell-cell interactions can be activated by extension forces (Fig. 6) (22, 23, 25, 31, 40). Thus, members of at least three gene families behavior similarly in response to hydrodynamic flow, which acts as an environmental signal.

The Als, Flo1, and Flo11 adhesin families are similar in the types of domains and their order within each sequence. However, these domains show no significant sequence similarity between the families. Each adhesin has a ligand-binding domain consisting of β-sandwich “Greek key” folds: Ig-like invasin domains in *C. albicans* Als adhesins, a PAI14 lectin domain in Flo1 family adhesin, and a fibronectin type III domain in Flo11p (10–12). Each adhesin also has Ser-Thr-rich TRs, an extended stalk region, and a covalent link to cell wall glucan through the remnants of a GPI anchor (13). Thus, the features common to these three families of adhesins include β-sandwich head domains, β-aggregation-prone sequences, Ser/Thr-rich TRs, glycosylated stalks, and GPI-derived cell wall anchors.

Ser/Thr-rich TRs in these adhesins do not show statistically significant sequence similarities (41). The TRs in Flo1p include 14 to 18 copies of a 45-residue sequence, each with a 5-residue core sequence with high β-aggregation potential (Fig. 1; see Table S1 in the supplemental material) (13, 20). The paralogs Flo5 and Flo9 contain similar repeat patterns, with similar β-aggregation-prone sequences (21). The repeats in Flo11p do not contain similar β-aggregation-prone sequences, but they are instead present in a more C-terminal region of the protein. There is a similar finding in Flo10, which also has a repeat profile different from that of the other Flo proteins and has β-aggregation-prone sequences near its C terminus (21). In Als proteins, the TRs include 3 to 36 copies of a 36-residue sequence. A single β-aggregation core sequence is present in well-conserved T domains (22, 42).

This similarity in adhesin architecture is reflected in similarities in the unfolding force-distance curves on protein unfolding in single-molecule AFM. Cell surface Flo1p (17) and Flo11p showed molecular characteristics similar to those of Als proteins. Each adhesin exhibited two types of force-distance profiles. Smooth low-force curves showed a single peak characteristic of molecular stretching and dissociation of the epitope from the antibody. High-force profiles showed a succession of sawtooth peaks characteristic of the unfolding of TRs. Therefore, these data imply that the midregions of the flocculins fold into multiple compact domains that can extend under tensile force, like the Als proteins (26). However, the contour distances between the peaks and the peak heights in Flo11p were more variable than in Als5p or Flo1p, perhaps reflecting the presence of several types of TRs in Flo11p (Fig. 1 and 6D). The adhesion force profiles showed similar maxima of ~200 to 300 pN and similar lengths of ~300 nm. Like that of Als and Flo1p, cell surface mapping of Flo 11p showed a high density of adhesins on the cell surface under the assay conditions used. Thus, there are similarities in force-distance responses to molecular stretching on AFM; however, we did not observe force-dependent clustering or increased exposure of the flocculins, phenomena seen in Als adhesins (23, 25, 33). The force-dependent increase in nanodomains was nevertheless apparent when monitored with thioflavin dyes (Fig. 5; Table 1; see Fig. S1 in the supplemental material) (22, 24). It is likely that this difference is due to the high surface density of the flocculins, which AFM detected at 500 to 730 molecules/µm^2^. This density is likely to be an underestimate, because biochemical or immunochemical values for Flo1p range from ~4 × 10^3^ to 2 × 10^5^ molecules/µm^2^ (13, 43). At such high densities, AFM is not sensitive enough to further investigate force-dependent clustering. Nevertheless, thioflavin fluorescence clearly documented increases in amyloid-like nanodomains under force.

The AFM cell-cell binding experiments confirmed that Flo11p acts as a homotypic binder, because adhesion between pairs of cells expressing Flo11p was extensive and that between Flo11p-expressing cells and EV-expressing cells was poor. This finding is consistent with coflocculation studies and monomer interactions in the crystal structure (12, 44). These cell-cell AFM assays also revealed that addition of methyl alpha-d-mannopyranoside did not affect the frequency or strength of binding. Therefore, the interaction was not dependent on a lectin-like interaction, again consistent with flocculation assays and with lack of mannose binding to the Flo11p head domain (12).

In contrast, the AFM and flocculation results for Flo11p clearly showed Ca^2+^-dependent activation. However, unlike that of Flo1p, the Flo11p head group does not bind Ca^2+^, so the cation dependence must be manifest in another region of the protein (1, 2, 12, 45). In both Flo1p and Flo11p, TRs are rich in Glu, so Ca^2+^ may well bind to δ-carboxyl groups and affect their conformation. Therefore, it may be that binding of Ca^2+^ to Glu residues in the TRs changes the conformation of TRs and exposes the Flo11p head domain to facilitate homotypic binding activity.

Of note, the flocculins were partially activated by force even in the absence of added Ca^2+^ (Fig. 2A and 3A). This partial activation led to increased flocculation on the subsequent addition of Ca^2+^ but was not sufficient to lead to macroscopic flocculation of the population (data not shown). Nevertheless, both the Ca^2+^-dependent and force-dependent partial activation may well depend on conformational remodeling of the proteins, leading to activation of the homophilic head domains (25, 46). Such a mechanism may be analogous to the Ca^2+^-induced conformational changes that activate cadherins (47).

### Fungal adhesins as force sensors.

Together, these results support a model in which the flocculins themselves respond to force, as does Als5p (23, 25). We have proposed that shear force unfolds the T domains in Als5p, exposes the aggregating core sequence, and leads to amyloid-like interactions with neighboring molecules to form cell surface nanodomains within minutes (16, 22–24, 40, 48, 49). The clustering of adhesins increases avidity, because there are localized patches with many arrayed adhesion molecules (22). The result is stronger adhesion.

The model extends to the flocculins because, like the Als adhesins, exposure to hydrodynamic shear potentiated flocculation, even in heat-killed cells. In each system, activation was inhibited by anti-amyloid dyes (Fig. 2, 3, and 7). Like Als5p, both flocculins mediate cell-to-cell aggregation that shows surface birefringence. Both are activated by laminar flow (20, 25). For both flocculins, vortex-induced shearing led to floc formation and increased surface binding of ThT (Fig. 5; Table 1; see Fig. S1 in the supplemental material). In the flocculins, as well as in Als5p, nanodomain formation followed 1 to 2 min of vortex mixing (Fig. 2D and 3B). In addition, the anti-amyloid treatment inhibited agar invasion, another force-dependent activity of flocculins (Fig. 4). Therefore, the evidence supports activation of all three adhesins through force-dependent clustering to form thioflavin-bright surface nanodomains (24, 25, 46).

These force-dependent interactions strengthen cell-to-cell bonding for all three adhesin families. In Als proteins, the added avidity complements ligand binding by the Ig-like invasion domains and hydrophobic-effect interactions through the TRs (22, 25). In the Flo1 family, these interactions are in addition to the PA14 domain lectin activity and proposed Ca^2+^-dependent carbohydrate-carbohydrate interactions (9). In Flo11p, the aggregative interactions must help activate the head domain homotypic interactions (12). In all three families, interactions are possible in *trans* between β-aggregating sequences in adhesins on opposing cells to form highly stable cell-cell bonds (22). Thus, force-dependent exposure and resulting aggregation would facilitate cell adhesion in each of these systems.

In summary, vortex mixing measurably and reproducibly activated fungal adhesins from three different gene families in two different yeasts. Bioinformatic searches and the presence of surface nanodomains in diverse fungal abscesses imply that such force-dependent aggregation of fungal cell surface proteins may be widespread (22, 50).

## MATERIALS AND METHODS

### Strains and media.

*S. cerevisiae* var. *diastaticus* (*MAT***a** *ura3 leu2-3*,*112 his4*) expressing Flo11p and the *flo11* deletion mutant strain (*MAT***a** *ura3 leu2-3*,*112 his4 flo11:URA3*) were kindly gifted by the late Anne Dranginis (St. John’s University). *S. cerevisiae* strain BX24-2B (*MAT*α *FLO1 gal1*) was purchased from the American Type Culture Collection (Manassas, VA). Cells used in invasion assays were grown in yeast extract-peptone-dextrose medium (YPD). Otherwise, cells were grown in adenine-enriched yeast extract-peptone-dextrose (YPAD) at 30°C and 170 rpm for 24 h.

### V5-tagged Flo11p.

Flo11-encoding DNA was copied from Yeplac+Flo11 (44) by PCR with primers that incorporated flanking sites for NotI and XhoI. Plasmid pJL1 was then cut with the same enzymes and ligated with the *FLO11* PCR product. The encoded protein includes the *S. cerevisiae* secretion signal, a V5 epitope, and 1,360 amino acids of Flo11 with its C-terminal GPI addition signal. The resulting plasmid, pJLFlo11, was transformed into ultracompetent *Escherichia coli* cells and amplified before transformation into *S. cerevisiae* strain W3031B (*MAT*α *leu2 ade2 ura3 his1 trp1*). Transformants were selected on synthetic complete (SC) medium lacking Trp. Expression of Flo11p was induced by growth in SC medium lacking Trp with galactose as a carbon source. For AFM experiments, cells were grown as described above overnight. Cells were harvested by centrifugation and resuspended in 10 ml of sodium acetate buffer containing 200 µM CaCl_2_.

### TANGO predictions.

Predictions of TANGO-positive protein sequences were done by uploading sequences to the website http://tango.crg.es. The sequences were tiled in windows of 500 residues with 100-residue overlaps. The pH was set at 5.5, and no N- or C-terminal protection was used. The temperature was 298.15 K, and the ionic strength was 0.02. A β-aggregation potential of >20% was considered positive (21).

Predictions of WALTZ-positive protein sequences were done by uploading the Flo11p sequence to the website http://waltz.switchlab.org/. The pH was set at 5.5 for both flocculins Flo1p and Flo11p, and the threshold was set for best overall performance.

### Flocculation assays.

Assays of flocculation mediated by Flo11p were carried out as previously described (2), with 2 × 10^7^ cells/ml prewashed with EDTA. This chelator is routinely used to dissociate adventitious flocs before assay. The added calcium is enough to titrate the EDTA and provide sufficient free Ca^2+^ to activate the flocculins (1, 2). Flo11p cells were washed three times and resuspended at 2 × 10^7^/ml in 20 mM sodium acetate buffer with 1 mM EDTA, pH 5.5. Flo1p-expressing cells were washed and resuspended to 6 × 10^7^/ml in 20 mM sodium acetate buffer with 200 µM EDTA, pH 5.5. Cells were vortex mixed or gently resuspended, and flocculation was initiated by addition of 670 µM CaCl_2_ for Flo1p-expressing cells and 330 µM CaCl_2_ for Flo11p-expressing cells unless stated otherwise. The suspensions were gently vortexed for 10 s at a low setting, and the optical density at 600 nm (OD_600_) was monitored at 5-s intervals in a Spectronic 21 D+ spectrophotometer. Flocculation rates were calculated as previously described (20). Unless stated otherwise, all assays were done with at least two independent cultures, each in triplicate. For visualization of flocs, 4-µl samples were placed on a slide and viewed with a 60× oil immersion objective.

### Dye inhibition.

Flo1p- and Flo11p-expressing cells were vortex mixed at 2,500 rpm or left quiescent for 5 min, and then ThS (200 µM) or CR (500 µM) was added to the cell suspension, corresponding concentrations of Ca^2+^ were added as mention above, and the OD_600_ was monitored.

### Confocal microscopy.

Confocal imaging was done with a Nikon Eclipse 90i microscope. A total of 10^8^ cells were stained with ThT (500 nM) in a final volume of 1 ml immediately after vortex mixing. The cells were vortex mixed at a low setting with the dye for 5 s to resuspend the dye, and then 4 µl of the suspension was placed on a glass slide for imaging. The stained cells were not washed prior to microscopy. The gain of the microscope was set at 7.75 with the phase at 162. The excitation wavelength was 408 nm with an emission detector wavelength of 450 ± 35 nm. Pictures were recorded at 2,048 by 2,048 pixels.

### Flow cytometry.

Flow cytometry was done with a BD Biosciences FACSCanto with an excitation wavelength of 405 nm and an emission filter wavelength of 450 ± 50 nm. A total of 10^8^ cells were placed into tubes measuring 12 by 75 mm with or without vortex mixing and then brought to a final concentration of 1 µM ThT or ThS in a total volume of 1 ml in the respective buffers as mentioned above. The cells were filtered with a 40-µm filter before analysis. A total of 20,000 cells were monitored for each assay.

### Staining protocols.

In general, we find that ThS effectively inhibits flocculation, whereas ThT stains surface amyloids well and is a less potent inhibitor (20, 24). Therefore, we used ThS as an inhibitor of flocculation and ThT to stain surface nanodomains. Stock concentrations of ThS and ThT were made with deionized water and filtered with a 0.2-µm filter. The concentration was then determined with a spectrophotometer by using Beer’s law and an extinction coefficient of 2.66 × 10^3^ liters/mol ⋅ cm in 100% ethanol. Staining of the adhesin-expressing cells was done with ThT and ThS (500 nM unless noted otherwise). Vortex-mixed or quiescent cells were slowly mixed with the added dye for 5 s. Aliquots (4 µl) of the cells were then added to a glass slide for microscopy.

### Single-molecule force spectroscopy.

Single-molecule measurements were performed at room temperature in sodium acetate buffer containing 200 µM CaCl_2_ with a Nanoscope VIII Multimode atomic force microscope (Bruker, Santa Barbara, CA). Cells were immobilized by mechanical trapping in porous polycarbonate membranes (Millipore) with a pore size similar to the cell size (51). After a concentrated cell suspension was filtered, the filter was gently rinsed with buffer, carefully cut (1 by 1 cm), and attached to a steel sample puck with double-sided tape, and the mounted sample was transferred into the AFM liquid cell while avoiding dewetting. Cells were first localized with oxide-sharpened microfabricated Si_3_N_4_ cantilevers (MSCT; Bruker Corporation), and the tip was replaced with a functionalized tip (see below). Adhesion maps on live cells were obtained by recording 32-by-32 force-distance curves in areas of 1 µm², calculating the adhesion force at rupture for each force curve, and displaying the value as a gray pixel. All force measurements were recorded with a contact time of 100 ms, an approach and retraction speed of 1,000 nm ⋅ s^−1^, and a maximum applied force of 250 pN.

AFM tips were functionalized with anti-V5 antibodies (Invitrogen) with polyethylene glycol (PEG)-benzaldehyde linkers as described by Ebner et al. (52). Briefly, cantilevers were washed with piranha solution, rinsed in ultrapure water (Elga), washed with chloroform and ethanol, and placed in a UV ozone cleaner for 15 min. They were then immersed overnight in an ethanolamine solution (3.3 g of ethanolamine in 6 ml of dimethyl sulfoxide [DMSO]), washed three times with DMSO and two times with ethanol, and dried with N_2_. The ethanolamine-coated cantilevers were immersed for 2 h in a solution prepared by mixing 1 mg of acetal–PEG–*N*-hydroxysuccinimide dissolved in 0.5 ml of chloroform with 10 µl of triethylamine, washed with chloroform, and dried with N_2_. Cantilevers were further immersed for 10 min in a 1% citric acid solution, washed with ultrapure water, and then covered with a 200-µl droplet of a solution containing anti-V5 antibody (0.2 mg/ml) to which 2 µl of a 1 M NaCNBH_3_ solution was added. After 50 min, cantilevers were incubated with 5 µl of a 1 M ethanolamine solution to passivate unreacted aldehyde groups and then washed with and stored in acetate buffer.

### Single-cell force spectroscopy.

Single-cell measurements were performed as previously described (17, 53). Briefly, single cells were attached to triangle-shaped tipless cantilevers (NP-O10 Microlevers; Bruker Corporation) coated with a bioinspired polydopamine wet adhesive by immersion for 1 h in a 10 mM Tris buffer solution (pH 8.5) containing 4 mg/ml dopamine hydrochloride (99%; Sigma) and drying under N_2_ flow. Single cells were then attached to cantilevers with a Bioscope Catalyst atomic force microscope (Bruker Corporation). To this end, 2 µl of cell suspension was added to 4 ml of sodium acetate buffer supplemented with 200 µM CaCl_2_ in a glass petri dish containing hydrophobic substrates covered with cell aggregates. The cantilever was brought into contact with an isolated cell for 3 min, and the cell probe obtained was then transferred over a cell aggregate for cell-cell force measurements. Force measurements were performed at room temperature (20°C) in buffer with a Bioscope Catalyst atomic force microscope (Bruker Corporation). The spring constant of the cantilever was ~0.06 N ⋅ m^−1^, as determined by the thermal-noise method. Multiple force-distance curves were recorded with a maximum applied force of 250 pN, a contact time of 1 s, and a constant approach-and-retraction speed of 1,000 nm/s. For each condition, the interaction forces of three yeast cell pairs from independent cultures were measured. For some experiments, 10 mM EDTA (Sigma Aldrich), 400 µM CaCl_2_ (Sigma Aldrich), 40 mg/ml methyl alpha-d-mannopyranoside (Sigma Aldrich), and 200 µM ThS (Sigma Aldrich) were added. Adhesion force and rupture length histograms were obtained by calculating the maximum adhesion peak and the last rupture distance for each force curve, respectively.

### Agar invasion.

Agar plates were prepared with 10 g/liter yeast extract, 20 g/liter peptone, 20 g/liter dextrose, and 20 g/liter agar. The mixture was autoclaved and then cooled while stirring on a hot plate. Appropriate amounts of filter-sterilized dye were added to the agar mixture, and then it was poured onto petri dishes. Yeast cells were grown in YPD overnight. Cells were then washed with deflocculation buffer, and 50 million cells in 50 µl of buffer were added to each plate. Cells were placed in a 30°C incubator for 2 weeks, after which the cells were washed off and images were obtained with a dissecting microscope.
