# Supplementary material for: Force Sensitivity in Saccharomyces cerevisiae Flocculins
Source: mSphere. 2016 Aug 17;1(4):e00128-16. doi: 10.1128/mSphere.00128-16 (PMC4989244; doi:10.1128/mSphere.00128-16)
Supplement: Table S1 [file sph004162130st3.pdf]

Supplemental Table S1: β-aggregation-positive sequences from *S. cerevisiae* adhesins

**Protein**	**β-aggregation sequence**	**β-aggregation (%)**	**Ile, Val, and Thr content (%)**
**Flo11p**	VTTVVSTTVV	80	90
	ITTTFV	53	83
	LVTTAVTTTVV	90	82
**Flo1p**	VYMYAGY	32	14
	TVIVI	89	100
	TIIVI (repeat 9 times)	89	100
	TVIVI (repeat 6 times)	89	100
	TVIVV	89	100
	TLVTVT	37	83
	AIVSTATVTV	48	70
	TVVTI	39	100
	TLVTVT	41	83
