# Supplementary material for: Force Sensitivity in Saccharomyces cerevisiae Flocculins
Source: mSphere. 2016 Aug 17;1(4):e00128-16. doi: 10.1128/mSphere.00128-16 (PMC4989244; doi:10.1128/mSphere.00128-16)
Supplement: Figure S1 [file sph004162130sf1.pdf]

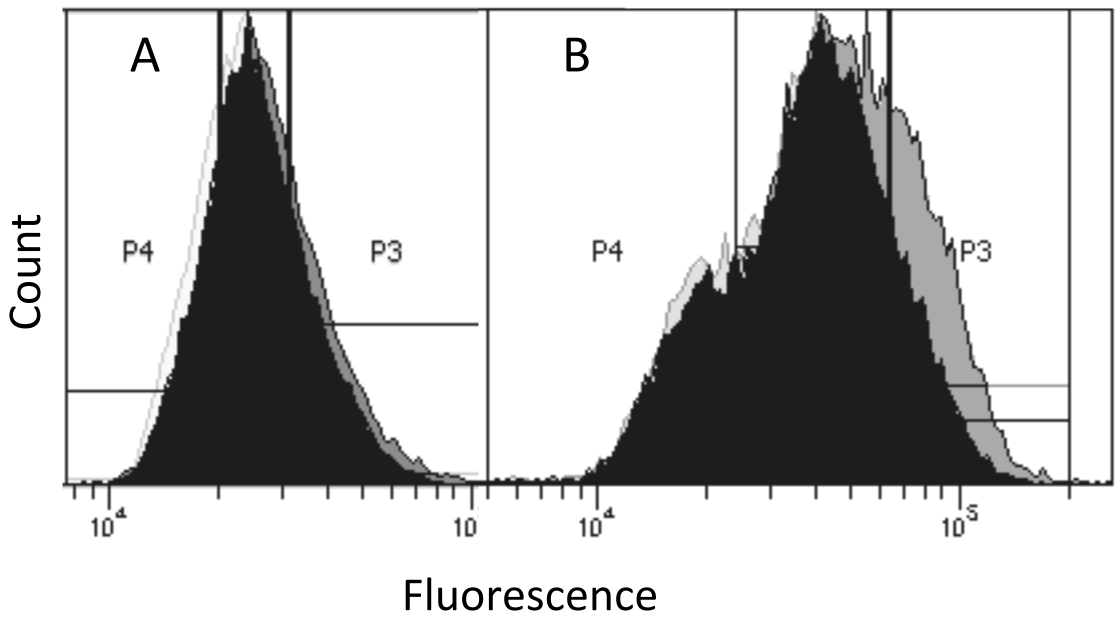

Supplemental Fig. S1. Overlays of ThS fluorescence of quiescent cell populations (black) or cells vortex-mixed for 5 min. (grey). A) Flo1-expressing cells; B) Flo11 expressing cells.
