# Supplementary material for: Force Sensitivity in Saccharomyces cerevisiae Flocculins
Source: mSphere. 2016 Aug 17;1(4):e00128-16. doi: 10.1128/mSphere.00128-16 (PMC4989244; doi:10.1128/mSphere.00128-16)
Supplement: Figure S2 [file sph004162130sf2.pdf]

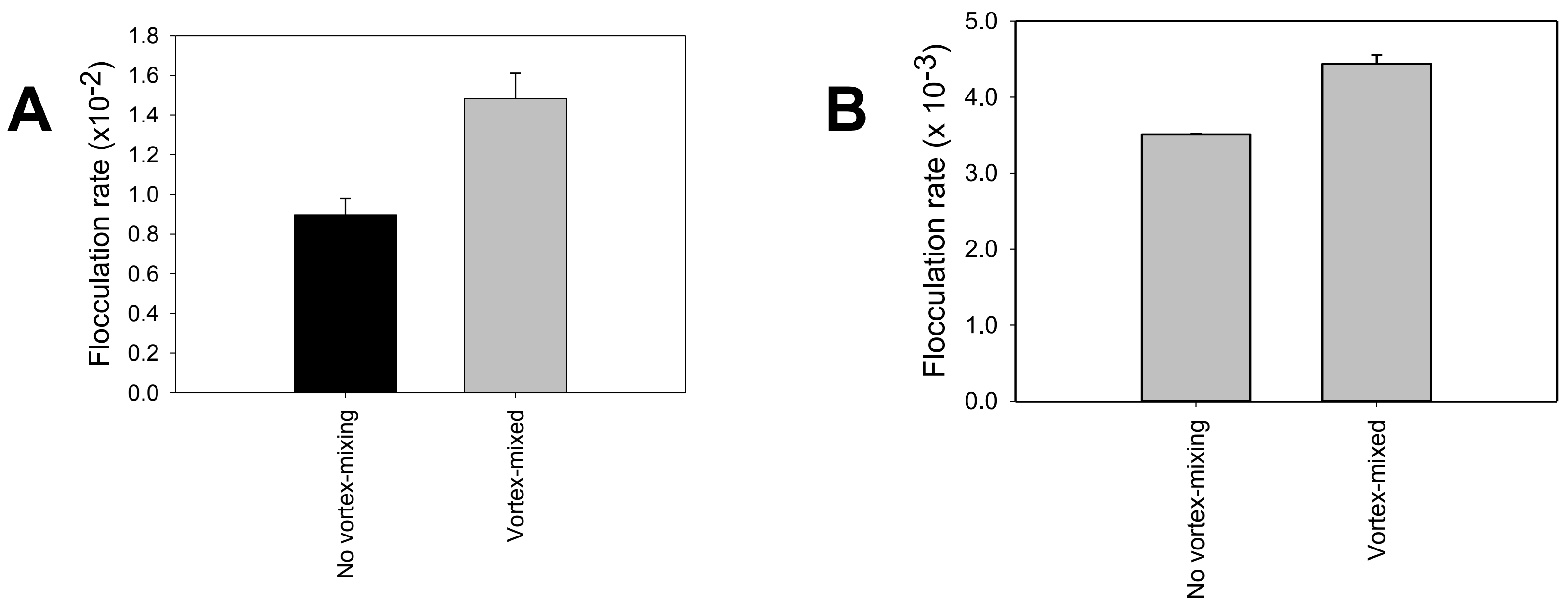

Supplemental Fig. S2. Effects of vortex mixing on heat-killed flocculin-expressing cells. (A) Effects of vortex-mixing on the flocculation rate of Flo11p-expressing cells after heat-killing. **(B)** Effects of vortex-mixing on the flocculation rate of Flo1p-expressing cells after heat-killing. Error bars represent standard deviation.
